# Heat Transfer and Residence Time Distribution in Plug Flow Continuous Oscillatory Baffled Crystallizers

**DOI:** 10.1021/acsomega.1c02215

**Published:** 2021-07-09

**Authors:** Naomi E. B. Briggs, John McGinty, Callum McCabe, Vishal Raval, Jan Sefcik, Alastair J. Florence

**Affiliations:** † EPSRC Continuous Manufacturing and Advanced Crystallisation Future Manufacturing Research Hub, Strathclyde Institute of Pharmacy and Biomedical Sciences, University of Strathclyde, 161 Cathedral Street, Glasgow G4 0RE, U.K.; ‡ EPSRC Continuous Manufacturing and Advanced Crystallisation Future Manufacturing Research Hub, Department of Chemical and Process Engineering, University of Strathclyde, 75 Montrose Street, Glasgow G1 1XJ, U.K.

## Abstract

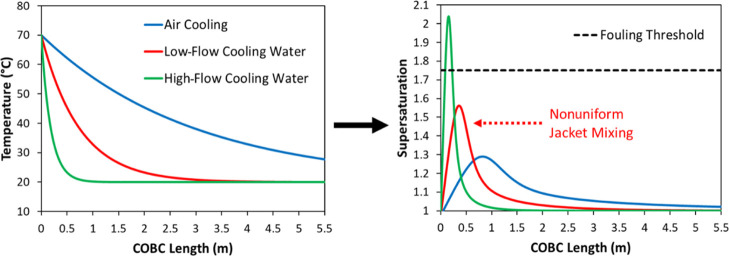

Heat transfer coefficients in a continuous oscillatory baffled crystallizer (COBC) with a nominal internal diameter of 15 mm have been determined as a function of flow and oscillatory conditions typically used under processing conditions. Residence time distribution measurements show a near-plug flow with high Peclet numbers on the order of 100–1000 s, although there was significant oscillation damping in longer COBC setups. Very rapid heat transfer was found under typical conditions, with overall heat transfer coefficients on the order of 100 s W m^–2^ K^–1^. Furthermore, poor mixing in the COBC cooling jacket was observed when lower jacket flow rates were implemented in an attempt to decrease the rate of heat transfer in order to achieve more gradual temperature profile along the crystallizer length. Utilizing the experimentally determined overall heat transfer coefficients, a theoretical case study is presented to investigate the effects of the heat transfer rate on temperature and supersaturation profiles and to highlight potential fouling issues during a continuous plug flow cooling crystallization.

## 1 Introduction

Crystallization is one of the major unit operations for the production of materials over a spectrum of industrial sectors including food, agrochemicals, pharmaceuticals, pigments, and dyes. Attention to platform performance, characterization, and process control is highly important due to the sensitivity associated with crystallization, that is, variables including mixing, flow, sheer, cooling rates, supersaturation, and antisolvent addition rates, dictating the physiochemical properties of the resulting product. Thus, for the regulation of material properties that can influence product performance such as taste, stability, bioavailability, and color, an understanding of influencing factors must be addressed to develop a well-controlled crystallization process.

Continuous manufacturing has been gradually adopted across many industrial sectors including food and chemicals, and more recently, it is of growing interest in pharmaceuticals.^1^ Crystallization has been traditionally performed in agitated tanks and other similar equipment, and some of these have been successfully adapted to continuous operation.^2^ Continuous crystallizations can also be performed in a variety of tubular platforms^3−9^ often somewhat simplistically referred to as plug flow reactors, including segmented flow and oscillatory flow platform devices. Benefits of operating within tubular platforms in contrast to tank systems may include better control of residence time distributions (RTDs) and local mixing, more efficient heat transfer resulting from a higher surface area to volume ratio, improved control over temperature profiles, and improved scale-up capabilities, hence reducing process development time from the laboratory to a manufacturing scale.^10−12^

Irrespective of platform choice, there are a number of key design features that will directly influence the control over a crystallization process.^13,14^ For example, when cooling crystallization procedures are implemented, heat transfer characteristics will dictate the rate at which supersaturation is generated. Poor heat transfer leads to temperature gradients resulting in supersaturation gradients within the bulk solution, thus potentially impacting processes including nucleation and growth. Conversely, if rapid heat transfer processes occur, care is needed to control bulk supersaturation levels to prevent large supersaturation spikes and associated undesirable nucleation events. Similarly, with antisolvent crystallizations, mixing can be the dominant parameter dictating supersaturation uniformity within a given crystallizer design.

Oscillatory baffled flow devices have been increasingly investigated for their applications as chemical reactors and crystallizers.^15−20^ The basic principle stems from a series of periodically spaced orifice baffles onto which an oscillation is applied. As the flow interacts with the baffles, eddies are created. This repeating oscillation cycle ensures strong radial mixing within the tubular vessels. An advantage of utilizing such a technology for crystallization is that mixing is independently achieved through the oscillations and thus decoupled from the overall net flow.^21^ The ability to extend the mean residence time, under plug flow conditions, at a similar mixing intensity, is advantageous to slow processes such as crystallization, where there is a need for longer residence times. The platform of interest within this study is the continuous oscillatory baffled crystallizer (COBC), and further information on relevant theory and operation can be found in the Supporting Information.

RTD experiments have been carried out previously in various setups of the oscillatory baffled flow equipment with internal diameters between 4 and 50 mm.^22−28^ These assessments suggest that operating conditions ensuring near-plug flow operation can be achieved in these systems and that superior heat transfer can be obtained when using oscillatory baffled flow conditions when compared to no oscillation and/or no baffles present. Studies measuring axial dispersion in oscillatory flow systems have utilized a wide range of geometries and operating conditions,^29−40^ and a summary of this literature can be found in the Supporting Information. Previous studies^23,27^ in other COBC geometries have shown that significant differences exist between liquid and solid axial dispersion at low mixing intensities but that under near-plug flow conditions, particles will experience similar dispersion characteristics to the bulk solution.

**Figure 1:**
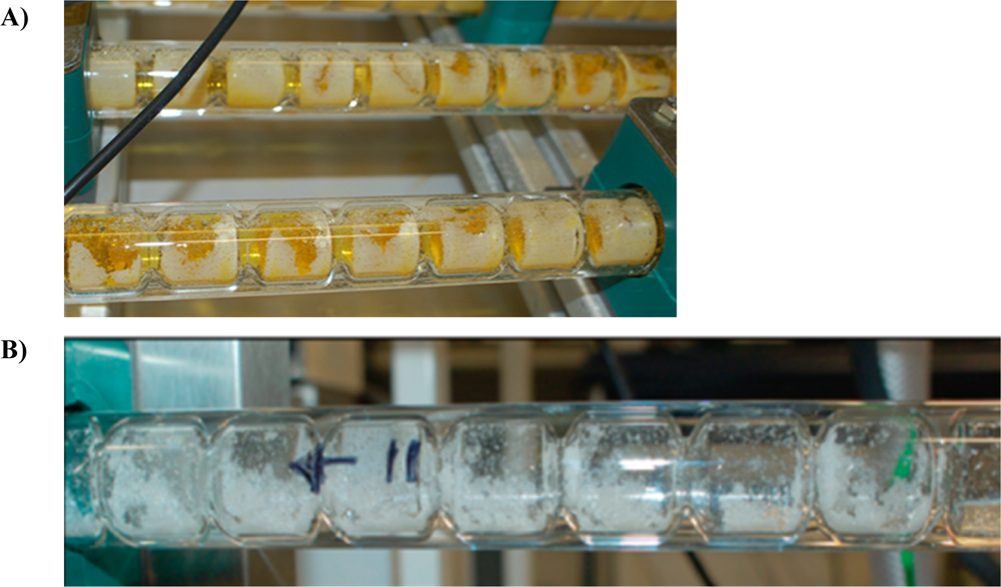
Examples of fouling upon the internal heat transfer surfaces of jacketed COBCs during (A) agrochemical^15^ and (B) l-glutamic acid^19^ crystallizations.

Heat transfer, in terms of Nusselt numbers, has been characterized in some oscillatory baffled flow geometries (inner tube diameters of 5 and 12 mm), with improvements reported when compared to their conventional tubular equivalents.^22,28,41^ However, the overall heat transfer coefficients have not been previously reported, and therefore, temperature profiles alongside individual COBC crystallizer sections have generally been unknown. Several studies^15,19^ using oscillatory baffled reactors for the application of continuous crystallization have reported fouling upon the internal heat transfer surfaces of the cooling jackets as a result of the cooling being too rapid (see Figure 1). However, the potential negative impact that enhanced heat transfer rates may have on continuous crystallization processes has not been previously addressed. Therefore, in addition to characterizing the heat transfer performance of oscillatory baffled crystallizers, we should use this understanding to better control heat transfer rates and prevent unwanted nucleation and/or fouling.

The COBC with a nominal internal diameter of 15 mm (DN15) is of particular interest due to its wide use in the academia and industry.^15,18−20^ This work therefore seeks to assess the performance of a DN15 COBC by quantifying overall heat transfer and liquid-phase axial dispersion under relevant processing conditions and assess the effects of heat transfer on local temperature and supersaturation profiles in order to improve the continuous crystallization process design and control.

## 2 Materials and Methods

### 2.1 Materials

Sodium benzoate (purity ≥99.0%, CAS 532-32-1) was purchased from Fluka. Deionized water was sourced on-site from a Thermo Scientific Barnstead RO water purification unit. Experiments were performed in an air-conditioned laboratory at 20 ± 2 °C.

### 2.2 Equipment

**Figure 2:**
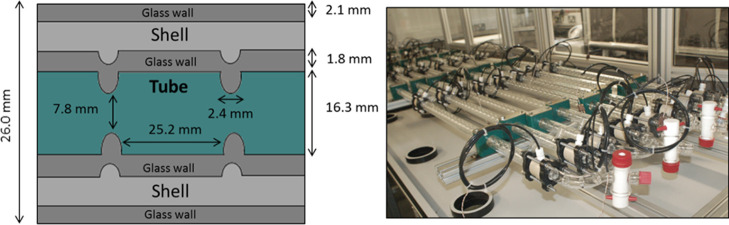
COBC tube geometry and setup. Schematic of a cross-section of a DN15 COBC straight (left) with dimensions indicated and a photograph of a COBC setup (right).

Experiments were completed in DN15 COBC (Nitech, U.K.) platforms, each consisting of a series of jacketed glass tubes (straights) and nonjacketed bends, ca. 70 and ca. 20 cm in length, respectively. Fluid oscillation was provided by a fluid-filled bellow unit controlled by custom electronics (housed in a control box). Peristaltic pumps (Watson-Marlow 520S) supplied the COBC with water stored in an independent stirred tank reactor. The geometry of the COBC straights and an example of the setup are shown in Figure 2.

### 2.3 Oscillatory Flow Background

Continuous oscillatory flow may be described by three dimensionless numbers: *Re*_o_, the oscillatory Reynolds number; *St*, the Strouhal number; and *Re*_*n*_, the net flow Reynolds number.^42,43^ These are governed by the following equations
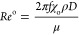
1
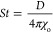
2
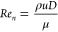
3where *D* = column diameter (m), χ_o_ = center-to-peak amplitude (m), *f* = frequency (Hz), ρ = density (kg m^–3^), μ = fluid viscosity (kg m^–1^ s^–1^), and *u* = mean velocity (m s^–1^). *Re*^o^ describes the intensity of oscillatory mixing applied within the tube, where 2π*f*χ_o_ is the maximum oscillatory velocity (m s^–1^). *St* is the ratio of the column diameter to the oscillation amplitude and is a measure of the eddy propagation inside each interbaffle zone. *St* is inversely proportional to χ_o_. *Ren*, the classic dimensionless number describing flows in pipes, is the ratio of inertial to viscous force within a flow.

Dominant oscillatory flow is required to maximize the effect of the eddy shedding cycle. That is, for the oscillatory flow to be effective, and to ensure that the flow is fully reversing, *Re*_o_ must be equal to or greater than *Re*_*n*_. A velocity ratio, ψ, was proposed to relate the oscillatory velocity to the net flow velocity and is described by^30^
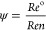
4

Peclet numbers (*Pe*) are often used to describe the deviation from the ideal flow behavior in tubular systems; the higher the value, the more plug flow-like the system is. The axial dispersion coefficient (*E*) is another term that can be used for flow identification, and the relationship between these two numbers can be expressed as follows^31^
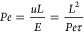
5

Pe numbers of approximately 1 suggest a back mixed flow behavior (CSTR-like), whereas *Pe* greater than 50 suggests a near-plug flow environment.^32^

### 2.4 Heat Transfer Equations

The overall heat transfer coefficient (*U*) is a measure of the overall ability of a series of conductive and convective barriers to transfer heat and is commonly applied to the calculation of heat transfer in heat exchangers. Considering that the COBC essentially takes the form of a shell-and-tube heat exchanger, the heat transfer performance of the DN15 COBC can be characterized by a set of overall heat coefficients for a range of flow and oscillation conditions.^41^ For a jacketed COBC straight, the overall heat transfer coefficient is determined by the following equation
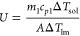
6where *m*_1_ = solution mass flow rate (kg s^–1^), *c*_*p*1_ = solution-specific heat capacity (J/kg °C), Δ*T*_sol_ = solution temperature change (°C), *A* = total heat transfer area (m^2^), and Δ*T*_lm_ = log mean temperature difference (°C).

Assuming that the jacket temperature is constant and there are no heat losses form the jacket, the temperature profile in the COBC can be modeled by utilizing the following differential equation
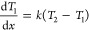
7where *T*_1_ = solution temperature (°C), *T*_2_ = jacket temperature (°C), *x* = COBC length (m), and *k* = differential equation constant (−). Assuming plug flow in the COBC, the differential equation constant (*k*) in the differential equation is given by
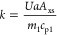
8where *a* = heat-exchange area per unit volume (m^–1^) and *A*_xs_ = cross-sectional area (m^2^). With the knowledge of the overall heat transfer coefficients and making certain assumptions, the differential equation can be solved either numerically or analytically to obtain the solution temperature (*T*_1_) at any COBC length (*x*). Depending on which assumptions are made, the temperature profile model may have three levels of depth. Level 1 assumes that the jacket temperature is constant and that there are no heat losses from the jacket. Level 2 assumes that the jacket temperature varies and that there are no heat losses from the jacket. Level 3 assumes that the jacket temperature varies and that there are heat losses from the jacket. Details of the differential equations and analytical solutions associated with each level of the temperature profile model can be found in the Supporting Information.

### 2.5 RTD Experimental Methods

**Figure 3:**
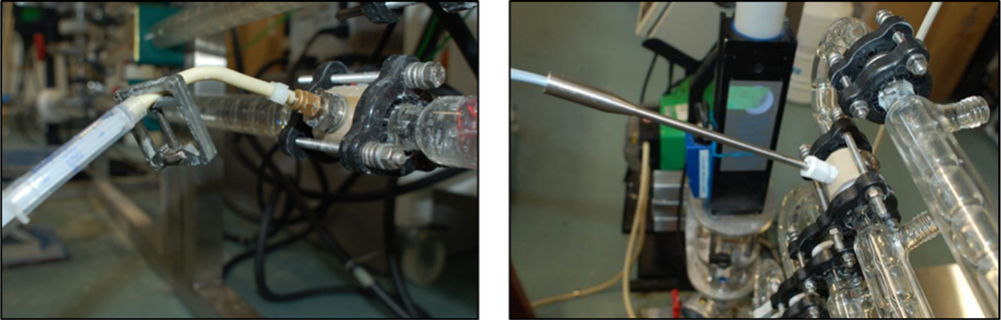
Injection port and in-line UV probe for RTD experiments. Photograph of the COBC setup showing the injection port (left); a syringe is connected to the port via Marprene tubing. Photograph of the UV transflectance probe fixed inline on the DN15 COBC via a connecting PEEK collar (right).

For RTD experiments, the COBC setup consisted of 22 straights and 11 bends. Tracer experiments were completed by manually injecting 2.3 mL of 0.5 % w/w sodium benzoate (Figure 3). The time evolution of the tracer was monitored using a Hellma UV transflectance probe with a 5 mm path length (Figure 3). Absorbance time data were collected using a Carl Zeiss MCS 501 UV spectrometer equipped with a CLD500 deuterium lamp (220–620 nm). Signals were recorded at 5 s intervals over a range of 190–320 nm using Aspect Plus software version 1.76. The λ_max_ of sodium benzoate was determined to be 226 nm, and a calibration model of *y* = 27.079*x* (*R*^2^ = 0.9988), where *y* is the absorbance (A.U) and *x* is the concentration (g/L), was used to calculate a concentration time response.

The UV transflectance probe placed at positions 1 at 2, ca. 4 m and 16 m away from the injection port respectively. Two flow rates, 50 and 200 g/min, were investigated corresponding to residence times of 15 min and 1 h, respectively. With regard to the COBC operating conditions, the oscillation frequency was either 1 or 3 Hz, and the oscillation amplitude ranged from 9 to 66 mm. Using the methods described elsewhere,^31^ the experimental data were fitted to a plug flow with the axial dispersion model exploiting the imperfect pulse technique. Ideally, the tracer absorbance would be measured at multiple positions simultaneously. However, due to the access of a single UV probe, multiple experiments were carried out in order to measure absorbance and subsequently calculate the concentration–time response at various distances downstream of the tracer injection port. The full set of operating conditions and a detailed explanation of the experimental data fitting can be found in the Supporting Information.

### 2.6 Heat Transfer Experimental Methods

**Figure 4:**
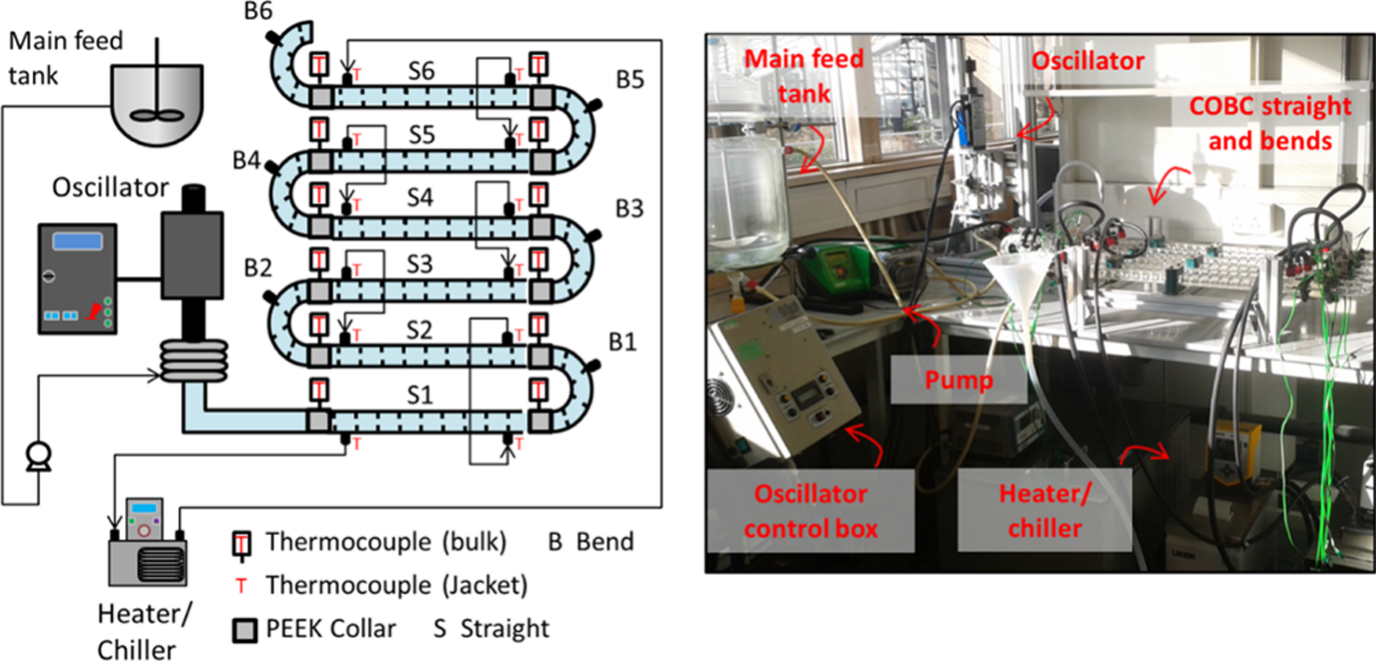
COBC setup for heat transfer experiments. Schematic (left) and photograph (right) showing the COBC setup used for heat transfer experiments operated in counter current (arrows indicate the direction of flow).

For heat transfer experiments, the COBC setup comprised six jacketed glass straights and six unjacketed glass bends (Figure 4). This smaller COBC setup was selected to simulate a typical single temperature zone of a larger COBC setup. Water coolant was circulated through the jackets in a counter-current manner by a heater/chiller (HC) (Lauda Eco RE 630S). The feed tank was controlled by an independent HC (VWR water bath 1187P) and internal flow rates were varied, alongside the variation in jacket flow rates. The feed solution was maintained at either 50 or 70 °C (to simulate an undersaturated feed solution for a cooling crystallization process), and the HC controlling the jacket was set to either 20 or 25 °C. The temperature of the solution inside the COBC, and that of the COBC jacket fluid, was measured at 12 corresponding points along the COBC, with the use of 24 temperature probes. Utilizing eq 6, overall heat transfer coefficients were calculated, and utilizing eqs 7 and 8, a temperature profile model was created (full details are given in the Supporting Information).

Two different modes of fluid flow were used for experiments: the traditional “single oscillation” where the tube side undergoes a net flow with superimposed oscillations (with a net flow only on the shell side) and the modified “double oscillation” where the tube and shell side both experience net flow with superimposed oscillations. Within each design, the bulk solution and jacket mass flow rates were varied to investigate the effect on the temperature profile. Oscillatory settings for both single-oscillation (tube side) and double-oscillation experiments (shell side and tube side) were set to a frequency of 1 Hz and an amplitude of 30 mm (trough-to-peak). The solution mass flow rate ranged from 50 to 200 g/min, whereas the jacket mass flow rate ranged from 50 to 2700 g/min. Full details of the experimental conditions are given in the Supporting Information.

### 2.7 Theoretical Case Study—Demonstrating the Effect of Rapid Heat Transfer on Temperature and Supersaturation Profiles in the Seeded Continuous Cooling Crystallization of Paracetamol

During seeded cooling crystallization processes, supersaturation levels should always remain within the metastable zone (MSZ) to control crystal growth and avoid unwanted nucleation. In addition to nucleation, there is also the need to avoid fouling of the crystallizer walls over time when operating at a steady state, which can be achieved by operating under the fouling threshold for that system. The concept of a fouling threshold has been demonstrated for oscillatory baffled crystallizers in a previous work.^44,45^ It should be noted that the fouling threshold is an empirical quantity that can be determined for specific crystallization conditions and vessel materials and the specific values are not a-priori known. If the MSZ width is narrow or the fouling threshold is low, then a lack of understanding around temperature (supersaturation) control can quickly result in uncontrolled nucleation and fouling of the crystallizer. Therefore, an accurate understanding of heat transfer along the crystallizer length is required to avoid operating in an undesired region.

This theoretical case study looks at the seeded cooling crystallization of paracetamol in a water/IPA (60/40 wt %) solvent mixture as a continuous process in the DN15 COBC. The model developed here applies the knowledge of the heat transfer performance of the DN15 COBC with a typical set of crystallization kinetics to determine the corresponding temperature and supersaturation profile associated with this crystallization process. With consideration of both the decrease in temperature and the increase in the total crystal mass with respect to the COBC length, the solution concentration (thus supersaturation) can be determined. The decrease in solution temperature, due to cooling, over the length of the COBC is described in eq 7 which is a differential equation with a constant given by eq 8. The increase in the total crystal mass, due to crystal growth, over the length of the COBC can be represented by the following equation
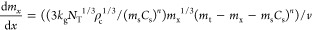
9where *m*_*x*_ = mass of API in the solid phase (kg), *x* = COBC length (m), *k*_g_ = growth rate constant (m s^–1^), *N*_T_ = total number of crystals (−), ρ_c_ = crystal density (kg m^–3^), *m*_s_ = solvent mass (kg), *C*_s_ = saturation concentration (kg/kg), *n* = growth order (−), *m*_t_ = total mass of API (kg), and *v* = velocity of the solution (m s^–1^). By simultaneously solving eqs 7 and 9, the solution concentration, and therefore the supersaturation, is known at any length along the COBC. The full details of the model can be found in the Supporting Information. In this case study, three different overall heat transfer coefficients were utilized with a typical set of experimental conditions in order to observe the effect heat transfer performance has on the temperature and supersaturation profiles.

## 3 Results and Discussion

### 3.1 RTDs and Axial Dispersion

**Figure 5:**
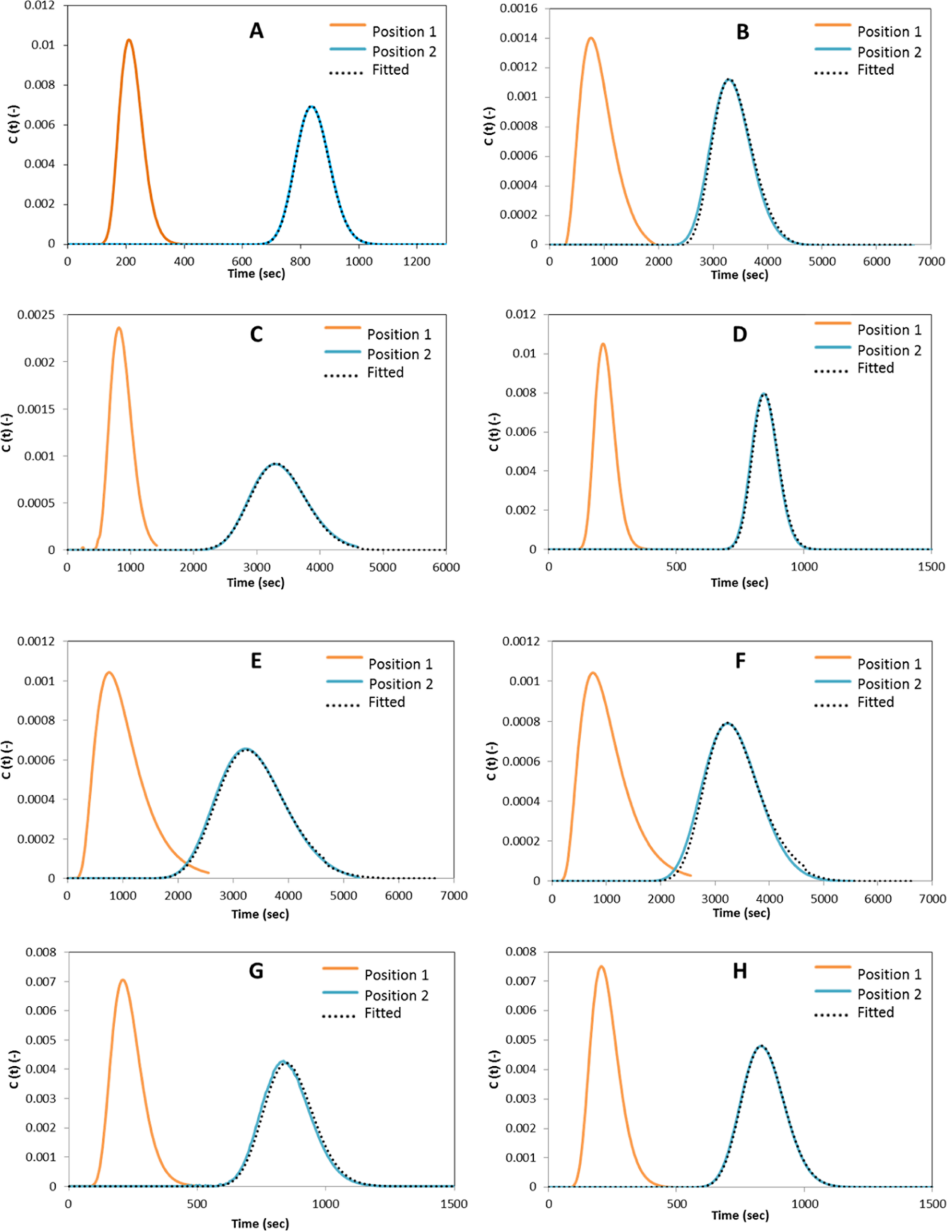
(A–H) Experimental data (orange color shows input and blue color shows response) and imperfect pulse model responses. Experimental data are in very good agreement with the fitted model response overlaid (dotted black).

The experimental RTD responses were analyzed using the imperfect pulse method.^31^ Representative examples of the model fitting procedure can be found in Figure 5. It can be observed that the fitted responses are in agreement with the experimentally measured profiles downstream, thus the axial dispersion model is found to be valid over the operating conditions tested.

The axial dispersion coefficients and Peclet numbers for each experiment were determined, and results can be found in Table 1. Over the experimental conditions investigated, *Pe* is shown to be above 100, which shows that deviations from plug flow in the liquid phase are insignificant. These results are in good agreement with previous studies,^24,25,27^ which utilized similar operating conditions with the same internal geometry, demonstrating that the DN15 COBC can operate within a plug flow regime when appropriate operating conditions are used.

**Table 1:** Experimental Conditions and Results from Imperfect Pulse Modeling

amplitude (mm)	frequency (Hz)	flow Rate (g/min)	*Pe*
38	1	50	150
38	1	50	550
38	1	200	650
38	1	200	1100
66	1	50	110
66	1	50	250
66	1	200	210
66	1	200	290
9	3	50	5500
9	3	50	4000
30	3	200	1800
30	3	200	1600
14	3	50	350
14	3	50	650

**Figure 6:**
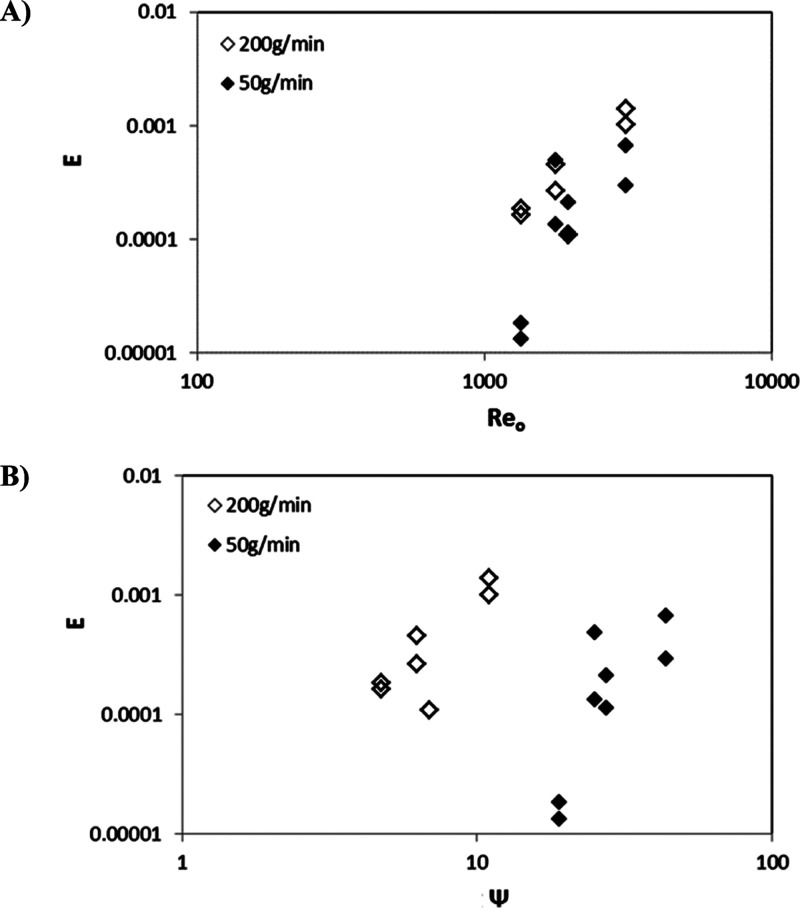
Axial dispersion coefficients plotted in terms of the dimensionless numbers describing oscillatory flows. (A) Axial dispersion coefficient *E* vs oscillatory Reynolds number *Re*_o_ and (B) axial dispersion coefficient *E* vs velocity ratio ψ for two different net flow rates.

The corresponding axial dispersion coefficients are further plotted in Figure 6 in terms of the dimensionless numbers describing oscillatory flows. The axial dispersion coefficient *E* increases as *Re*_o_ and ψ are increased, as expected. Increasing the oscillatory Reynolds number *Re*_o_ (and thus the velocity ratio, ψ, at a fixed net flow rate) corresponds to increasing oscillation intensity and thus more effective local mixing, hence increased axial dispersion, which shows strong power-law dependencies on both *Re*_o_ and ψ. It can also be seen from Figure 6 that the axial dispersion coefficient depends only little on the net flow rate (and thus *Re*_n_) under the conditions investigated here. These observed trends agree well with the previous literature,^24,25,27^ which used similar operating conditions with the same DN15 COBC geometry. Therefore, the oscillatory Reynolds number *Re*_o_ is an appropriate scaling parameter on which comparison with other conditions and geometries can be based.

### 3.2 Oscillation Damping

**Figure 7:**
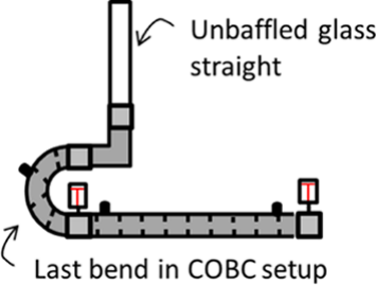
Schematic showing the setup for oscillation calibration.

**Figure 8:**
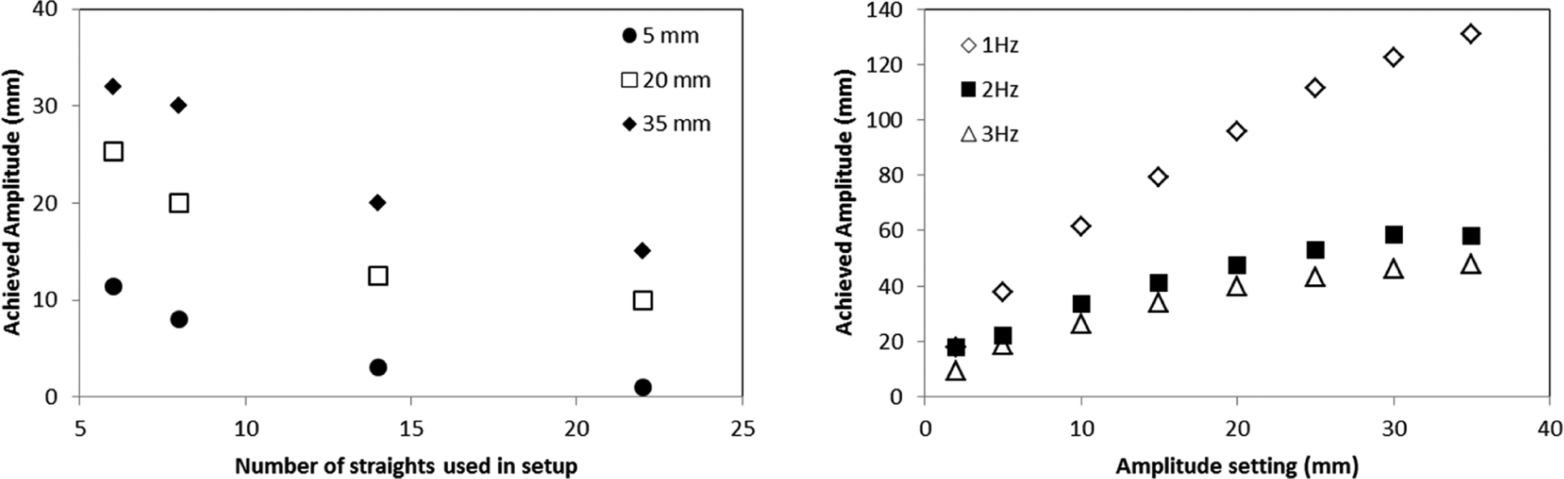
Calibration of oscillation amplitude in a DN15 COBC at various setup lengths. Graph (left) illustrating a decrease in amplitude as the number of straights in the COBC system is increased (fixed frequency of 3 Hz) and a graph (right) illustrating the achieved amplitude with increased frequency (measured in a fixed setup of 12 COBC straights).

Observations made during this study suggested discrepancies in the achieved fluid oscillation amplitudes when using the same input setting on the control box but at a different setup length. A calibration was carried out by positioning an unbaffled glass straight vertically at the outlet of the COBC (Figure 7), filling it partially with water and visually monitoring the displacement at various control box settings without net flow. The results of this calibration (Figure 8) showed a significant oscillation damping effect with respect to the setup length.

Oscillation damping along the COBC is thought to be due to the oscillatory wave losing energy (overcoming friction at the vessel walls and during fluid contact with the baffles for example) as it dissipates through the system. Oscillation damping has potentially significant implications for flow control, particularly for larger volume/length crystallizers. While oscillation damping did not have an impact on the measurement of accurate RTD curves, it raises interesting questions for crystallization in terms of the impact of variable oscillatory mixing. For example, damping of oscillations could be advantageous in situations where more intense mixing is required, at the start of the process, to induce nucleation readily. Oscillation damping may also have application in the control of shear-sensitive systems, as a reduction in shear, due to reduced mixing intensity, along the length of the system could minimize the formation of fines. Conversely, oscillation dampening could also be problematic as more intense mixing may be needed to suspend the crystals as they grow to larger sizes. In such a situation, this damping effect would be unfavorable. A greater understanding of the change in mixing intensity along the tubular crystallizer is required to permit accurate control over critical process parameters. These observations also highlight the need to develop better engineering solutions for the oscillation control. Options include operating the system with a double-acting piston unit and operating several COBCs in series to ensure control and sufficient oscillation intensity at the end of the COBC system. The results presented here assume that no oscillation damping occurs.

### 3.3 Heat Transfer Coefficients and Temperature Profile Modeling

**Figure 9:**
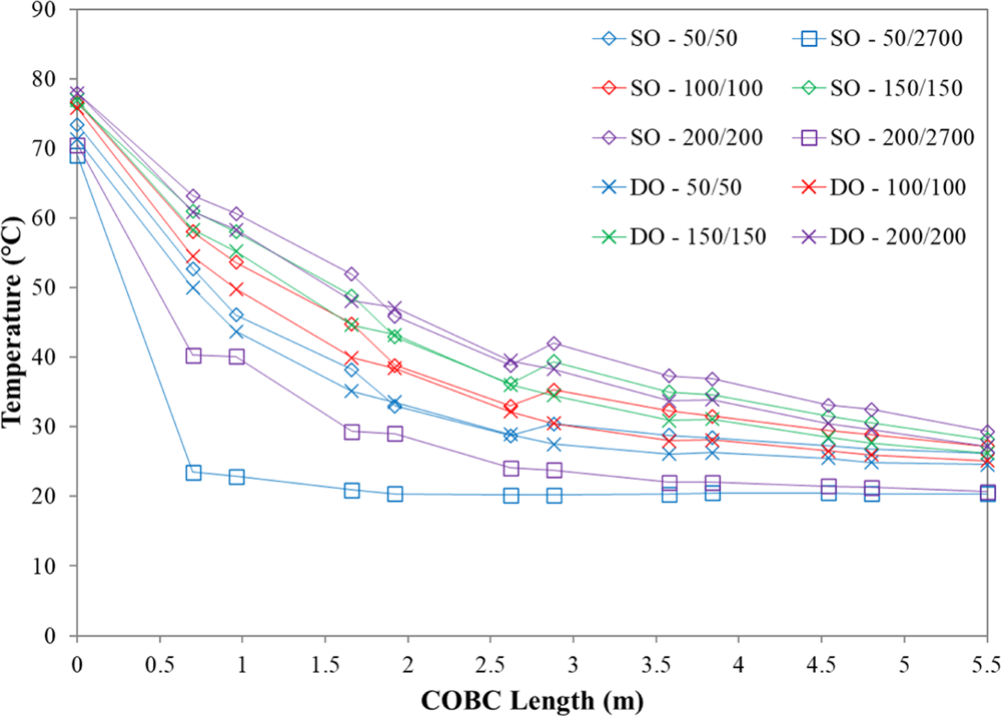
Representative experimental temperature data for a subset of single-oscillation and double-oscillation experiments. The data have been connected by lines as a guide only (lines do not represent the actual profile).

**Figure 10:**
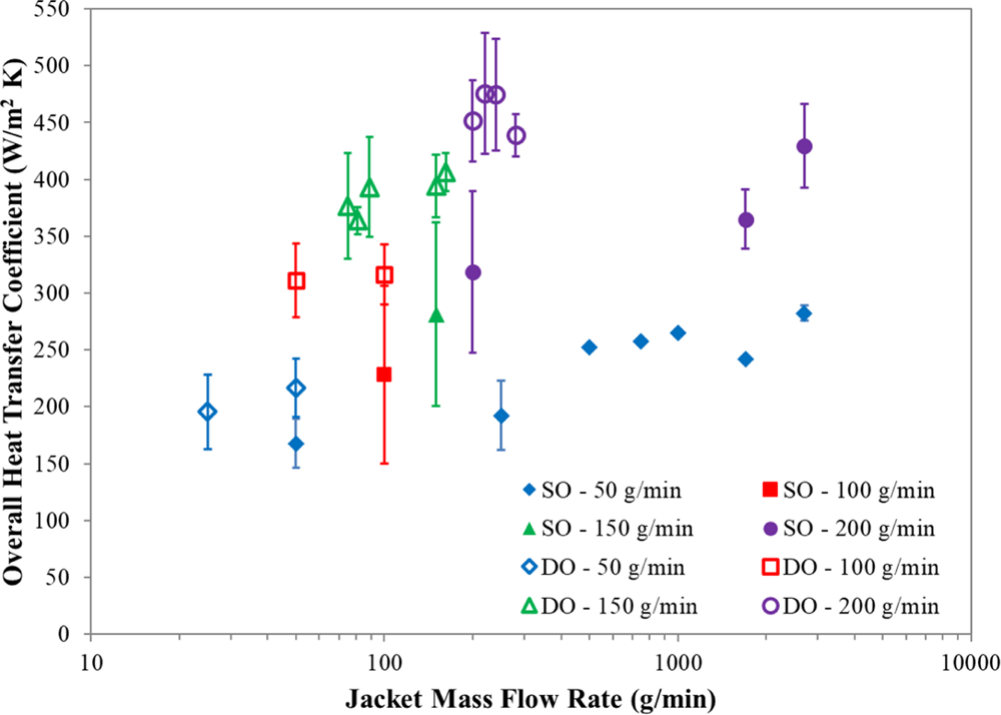
Overall heat transfer coefficients in a DN15 COBC. Graph showing overall heat transfer coefficients with respect to the jacket mass flow rate with and without oscillation of the shell fluid.

To assess solution temperature profiles along the crystallizer length, a series of experiments were carried out and the temperature data were acquired with respect to the COBC length. Representative experimental temperature data for a subset of single-oscillation and double-oscillation experiments can be found in Figure 9. The overall heat transfer coefficients (*U*) were calculated from the experimental temperature data using eq 6. Only *U* values which fulfilled two criteria were included in the final data set. The first criterion was that the solution must be cooled by at least 1 °C in the corresponding experiment. This criterion was imposed to minimize the influence of the error in the experimental temperature measurements. The second criterion was that for each set of conditions (oscillation setup, solution mass flow rate, and jacket mass flow rate), the *U* value must lay within 1.75 standard deviations of the mean *U* value. This criterion was imposed to remove statistical outliers. The final overall heat transfer coefficient data set is plotted with respect to the jacket mass flow rate in Figure 10.

For both the single- and double-oscillation setups, experimental temperature profiles and the overall heat transfer coefficients demonstrate that increasing the ratio of the jacket mass flow rate to the solution mass flow rate results in the solution cooling at a faster rate, as expected. Furthermore, the double-oscillation setup results in the solution cooling at a faster rate than the single-oscillation setup. This is also to be expected as the oscillation of the shell fluid increases the mixing within the shell, thus facilitating heat exchange.

**Figure 11:**
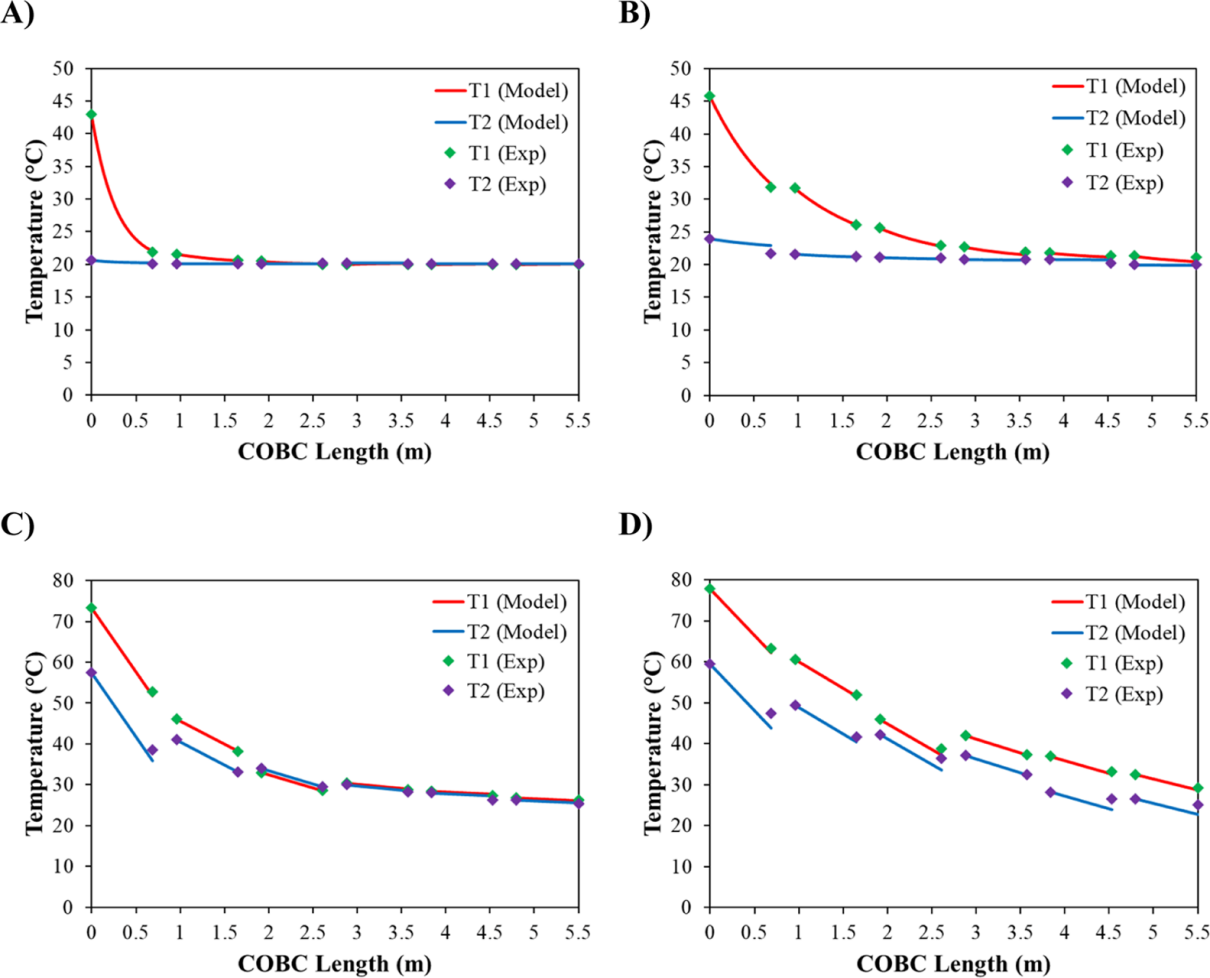
Experimental data points with modeled temperature profiles. (A) SO—50/2700, (B) SO—200/2700, (C) SO—50/50, and (D) SO—200/200.

In order to assess the accuracy of the overall heat transfer coefficients and the validity of the temperature profile model, experimental responses were compared to model predictions. Figure 11 shows an example of this over four experiments, SO—50/2700, SO—200/2700, SO—50/50, and SO—200/200. This example utilized level 2 of the temperature profile model (full details are given in the Supporting Information) which assumes that although the jacket temperature varies, there are no heat losses from the jacket to the surrounding air. It can be noted that the modeled temperature profiles are a good fit to the experimental temperature data points for the high-jacket mass flow rate experiments (Figure 11A,B). However, when a low jacket mass flow rate is used, predicted and measured responses become less similar (Figure 11C,D). These results suggest that at high jacket mass flow rates, the jacket is sufficiently mixed, whereas at lower jacket flow rates, the mixing within the shell becomes less efficient. This hypothesis was tested with the full results being shown in Section 3.4.

### 3.4 Cooling Jacket Flow Separation

**Figure 12:**
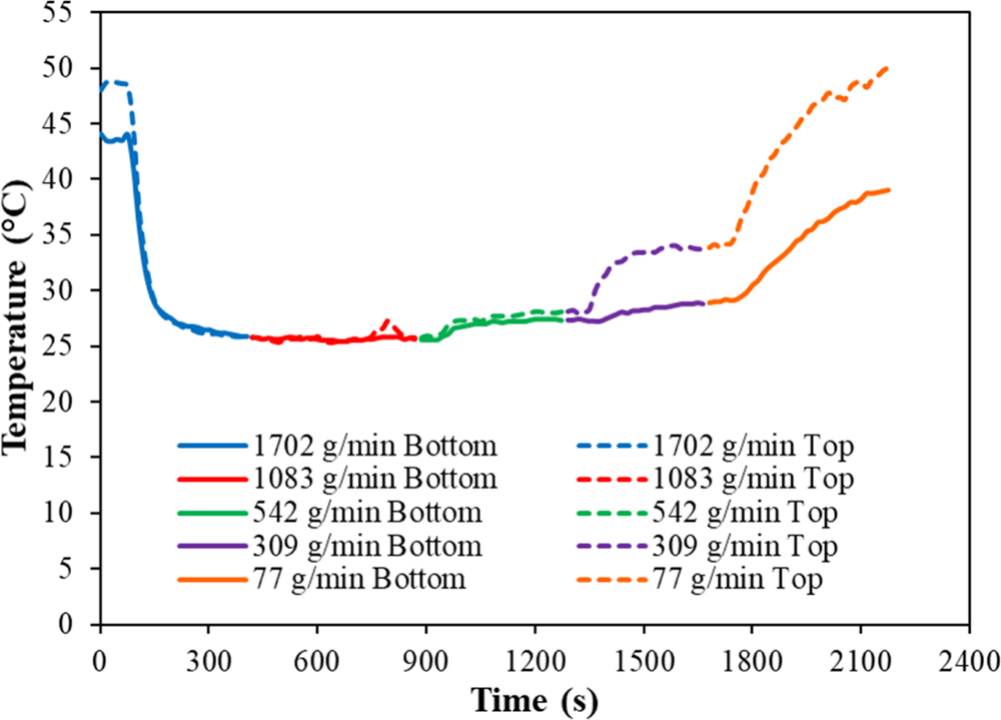
Jacket mixing test. Graph showing the temperature on the top and bottom of a straight at various jacket mass flow rates with a constant solution mass flow rate of 169 g/min.

To assess the hypothesis that mixing within the jacket shell is insufficient for good heat exchange at low jacket flow rates, an experiment was designed where temperatures were monitored at the top and bottom of the cooling jacket during a continuous cooling experiment. Here, the solution mass flow rate was kept constant at 169 g/min, while the jacket mass flow rate was lowered from 1702 to 77 g/min in a stepwise fashion (in order to measure the difference in temperature between the top and bottom of the jacket for each flow ratio). A single-oscillation setup with oscillation conditions of 1 Hz and 30 mm was used. Figure 12 shows the results obtained to assess mixing within the shell. Here, higher jacket mass flow rates (542–1702 g/min) show a negligible temperature difference between the top and bottom of the jacket. However, when the jacket mass flow rate lowers to 309 g/min, the temperature difference between the top and bottom of the jacket increases to ca. 5 °C. This temperature difference further increases to ca. 11 °C when the jacket mass flow rate lowers to 77 g/min. These results therefore illustrate flow separation within the shell. The top of the jacket is significantly warmer than the bottom, indicating a lower flow rate at the top of the shell when compared to the bottom. Therefore, the mixing in the jacket becomes poor for low ratios of the jacket mass flow rate to solution mass flow rate, which causes the inaccuracy of the temperature profile model under these conditions.

### 3.5 Effects of Rapid Heat Transfer on Temperature and Supersaturation Profiles

**Figure 13:**
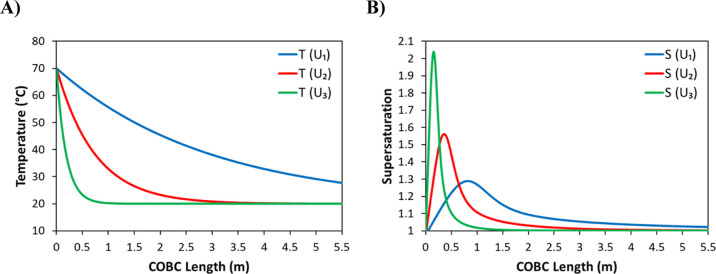
(A) Calculated temperature profiles and (B) calculated supersaturation profiles corresponding to three different overall heat transfer coefficients (see Table 2).

The temperature and supersaturation profiles obtained by using three different overall heat transfer coefficients were calculated using the heat transfer model and the seeded cooling crystallization model and are shown in Figure 13. The smallest value of the overall heat transfer coefficient would be the result of utilizing air cooling, the median value, the result of utilizing a low flow rate of cooling water, and the largest value, the result of utilizing a high flow rate of cooling water. For these modeled experiments, the values of the key constants, in addition to the three overall heat transfer coefficients, can be found in Table 2.

**Table 2:** Values of the Key Constants and Overall Heat Transfer Coefficients Used in the Calculation of the Temperature and Supersaturation Profiles

constant	symbol (unit)	value
growth rate constant	*k*_*g*_ (m s^–1^)	1 × 10^–6^
growth order	*n* (−)	1.5
seed loading	*m*_*x*0_ (wt %)	1
initial diameter of seeds	*d* (μm)	20
overall heat transfer coefficient (1)	*U*_1_ (W/m^2^ K)	25
overall heat transfer coefficient (2)	*U*_2_ (W/m^2^ K)	100
overall heat transfer coefficient (3)	*U*_3_ (W/m^2^ K)	400

The plots in Figure 13 show that the magnitude of the overall heat transfer coefficient has a profound effect on the temperature and supersaturation profiles during a seeded cooling crystallization process. Considering the results from a previous study on the seeded cooling crystallization of paracetamol,^45^ the typical fouling threshold at glass surfaces would be around 1.75. Using this threshold, fouling can be expected within the first few tens of centimeters of the crystallizer when using a high flow rate of cooling water (overall heat transfer coefficient of 400 W/m^2^ K) where the heat transfer is most efficient (see the green line in Figure 13 B). Furthermore, there would be little residual supersaturation left after the first meter of the crystallizer length, and therefore, the corresponding volume would not be efficiently utilized. The case of the low flow rate of cooling water (overall heat transfer coefficient of 100 W/m^2^ K) would be more favorable, avoiding the fouling threshold, but there would be cooling jacket flow separation (see Section 3.4), so nonuniform cooling alongside the inner tube circumference would occur without further equipment modification. Therefore, a much slower heat transfer rate corresponding to air cooling would be more appropriate to control continuous seeded cooling crystallization under the plug flow conditions examined here.

In the case of unseeded crystallization, there would be even larger supersaturation spikes as those shown in Figure 13B, and there is a clear issue with a potential fouling risk. For the case of seeded crystallization considered here, it would be possible to increase seed loading to manage local supersaturation spikes. However, seed loading is typically constrained by the target crystal size distribution required and thus not necessarily adjustable to the extent that may be required to alleviate abruptly steep temperature profiles alongside cooling crystallizer sections.

There is a wider point to be made, although it may seem somewhat counterintuitive, that it is not necessarily good to have high heat transfer coefficients for plug flow cooling crystallizer units. This is because cooling crystallizers generally require gradual, well-controlled temperature profiles. In order to achieve such profiles within individual cocurrent or counter-current cooling sections, such as those used in COBCs investigated here, smaller heat transfer coefficients are desirable. Otherwise, the inlet stream temperature exponentially decreases toward the outlet temperature within a fraction of the cooling section length and the rest of it is at a nearly constant temperature (Figure 13A), leading to an unfavorable temperature profile across the section. Alternatively, very small differences in inlet and outlet temperatures would be needed for higher heat transfer coefficients, leading to very small temperature changes in each section, which would require a large number of sections, which becomes rather impractical. Instead of high heat transfer coefficients as such, what is needed is to be able to access a suitable range of heat transfer coefficient values depending on the number of cooling sections and residence time required. To summarize, in order to control supersaturation profiles and mitigate potential fouling along a continuous plug flow seeded cooling crystallizer, a much wider range of overall heat transfer coefficients may be needed than that provided by the platform investigated here.

In general, it is desirable to move away from inducing a step-change cooling profile in continuous plug flow cooling crystallization and allow for the control of an optimal cooling profile, for example, linear or parabolic, along the entire crystallizer length. This would make it more amenable for delivering the desired supersaturation profile required for well-controlled crystallization. One possible approach is to use air cooling in the DN15 COBC, in which the case study would result in a smoother temperature profile. Air cooling has been used for continuous crystallization processes in other tubular devices,^46^ resulting in more linear temperature profiles, which demonstrates that this cooling method could be successfully implemented. Another approach for avoiding step-change cooling has been demonstrated by the Cambridge Reactor Design Rattlesnake multiorifice COBC which has four zones with a double-shell heat exchanger^47^ that enables smoother temperature profiles to be delivered across the entire crystallizer length.

## 4 Conclusions

DN15 COBC achieves low axial dispersion and operates with near-plug flow under a wide range of operating conditions. Variations in operating conditions (*Re*_o_ and *Re*_n_) result in significant changes to axial dispersion. Increasing *Re*_o_ and the velocity ratio results in a power-law increase in axial dispersion coefficients. Nevertheless, there is a wide operating window in which reasonable plug flow conditions can be achieved and, as such, the platform has good potential to support plug flow-based processes. This study also provides the demonstration of the significant oscillation damping that occurs over extended lengths of a COBC system, which is the phenomenon that needs to be accounted for when designing crystallization processes.

This work characterized the heat transfer performance of a DN15 COBC through the determination of the overall heat transfer coefficients over a range of flow and oscillation conditions. Based on this, a heat transfer model has been implemented, which accurately predicts the temperature profile across individual COBC straights. The ability to accurately model the temperature profile in the COBC is crucial for the design and control of a continuous plug flow cooling crystallization as the resulting crystal properties are directly influenced by the supersaturation profile along the crystallizer, while the fouling threshold should be avoided along the whole crystallizer length. Heat transfer has been quantified within the DN15 COBC with overall heat transfer coefficients over jacketed straights and unjacketed bends being shown to be in the range of 150–500 (water cooled) and 10–50 (air cooled) W/m^2^ K, respectively.

In addition to characterizing heat transfer performance, this work has identified the limitations of the current cooling system of the COBC platform investigated. First, the COBC is typically operated with a very high flow rate in the cooling jacket, which results in the rapid cooling of the crystallization solution and may result in unfavorable temperature profiles where the temperature very quickly decays over a short length, leading to a poorly controlled crystallization process and potential crystallizer fouling. In an effort to obtain a more gradual cooling process, the mass flow rate in the jacket was substantially lowered. Although this approach did produce a smoother temperature profile, it resulted in poor mixing throughout the jacket. Poor mixing in the jacket is a major issue as it may result in a large temperature difference between the top and bottom of the straights in the COBC, which could result in fouling at the cold bottom and/or crystal dissolution at the warm top. Using the heat transfer model and a theoretical case study of the seeded cooling crystallization of paracetamol, we showed that a wide range of overall heat transfer coefficients need to be accessible in order to control temperature and supersaturation profiles and avoid potential fouling along a continuous plug flow cooling crystallizer.

## Supporting Information Available

COBC theory and operation; residence time distribution measurements in the literature; full description of experimental conditions and data fitting for residence time distribution studies; full experimental conditions and temperature data for heat transfer studies; full description of temperature profile model; and temperature and supersaturation profile modeling for the crystallization process design in the COBC (PDF)
