# Supplementary material for: Heat Transfer and Residence Time Distribution in Plug Flow Continuous Oscillatory Baffled Crystallizers
Source: ACS Omega. 2021 Jul 9;6(28):18352–63. doi: 10.1021/acsomega.1c02215 (PMC8296600; doi:10.1021/acsomega.1c02215)
Supplement: Supplementary file 1 — ao1c02215_si_001.pdf [file ao1c02215_si_001.pdf]

Supporting Information

Heat Transfer and Residence Time Distribution in Plug Flow Continuous Oscillatory Baffled Crystallisers

Naomi E. B. Briggs,[†] *John McGinty,*[‡] *Callum McCabe,*[‡] *Vishal Raval,*[†] *Jan Sefcik**[,‡] *and Alastair J. Florence**[,†]

[†]EPSRC Continuous Manufacturing and Advanced Crystallisation Future Manufacturing Research Hub, Strathclyde Institute of Pharmacy and Biomedical Sciences, University of Strathclyde, 161 Cathedral Street, Glasgow, G4 0RE, U.K.

[‡]EPSRC Continuous Manufacturing and Advanced Crystallisation Future Manufacturing Research Hub, Department of Chemical and Process Engineering, University of Strathclyde, 75 Montrose Street, Glasgow G1 1XJ, UK.

KEYWORDS

Residence time distribution, heat transfer, continuous oscillatory baffled flow, plug flow, oscillatory flow, axial dispersion, continuous crystallisation

1. Continuous Oscillatory Baffled Crystalliser Theory and Operation

The basic principle of the continuous oscillatory baffled crystalliser (COBC) stems from a series of periodically spaced orifice baffles on to which an oscillation is applied. As the flow interacts with the baffles, eddies are created. This repeating oscillation cycle ensures strong radial mixing. Figure S1 illustrates the flow interactions inside the COBC.

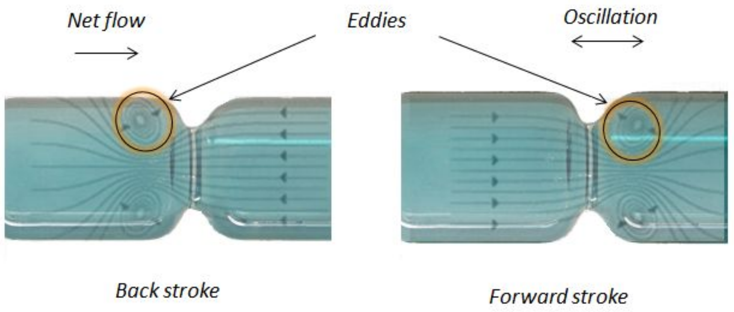

Figure S1. Flow interaction within baffled tubes. Photograph of baffles with flow patterns superimposed. Eddies are generated due to oscillatory flow.

The ability to extend the mean residence time, under plug flow conditions, at a similar mixing intensity, is advantageous to slow processes such as crystallisation, where there is a need for longer residence times. For example, a 25 m DN15 COBC operating under oscillatory conditions of 1 Hz, with a 30 mm amplitude and a flow rate of 50 g min^{-1} has a residence time of approximately 90 minutes, where Re_o = 1500 and Re_n = 65. To achieve comparable mixing intensity in a non-baffled and non-oscillated tubular system of the same dimensions, the net flow rate would need to be increased from 50 g min^{-1} to 1200 g min^{-1}, which would result in an approximate residence time of only 4 minutes i.e., 5% of oscillatory baffle flow.

When utilizing oscillatory flow technology there are namely three variables that may be selected for operation for a fixed reactor length, oscillatory frequency, oscillatory amplitude and net flow rate.[1, 2] Selection of optimal conditions will be dictated by the process, for example oscillatory conditions will be selected which are capable of suspending material at completed desupersaturation, also for a fixed length of a system a particular residence time is required, to achieve sufficient growth and size of the resulting particle. To achieve this various oscillatory conditions, residence times and net flow rates will be employed depending on the compound of interest investigated and associated crystallisation kinetics. Thus, a range of operating conditions within the system needs to be studied to provide an operation design space for crystallisation applications.

2. Residence Time Distribution Measurements in Literature

There have been many studies measuring residence time distributions (RTDs) in oscillatory flow systems with a wide range of geometries and operating conditions. From these studies it has been found that ideal operating conditions include working within velocity ratios between 2 – 10 and $Re_o \geq 100$. Table S1 shows a summary of these previous reports on RTD behaviour within oscillatory flow systems. The range of geometries makes it difficult to make appropriate comparisons.

Table S1. Previous RTD studies of oscillatory baffled mixing technologies.[3-14]

Tube ID (mm)	System length (m)	BS_r	Baffle ID (mm)	α	Re_n	Re_o	St	Results
23	0.67	1.5	13	0.44	110	100 - 3300	0.3 - 9	*D/uL:* 0.025 - 0.128
51	2.5	1.5	32	0.61	40, 106	160 - 2500	0.8, 2	1/*Pe*: 0.8 - 2
25	1.08	1.5	16	0.34	128	40 - 3600	0.35 - 4	*D/uL:* 0.030 - 0.10
25	6.3	1.5	16	0.34	106 - 3400	100 - 750	0.4 - 3	*D/uL:* 0.003 - 0.012
23.5	2.9	1.5	12		95, 127, 190, 252	0 - 2500	0.4 - 3.8	tanks-in-series model
40	25	1.8	32	0.21	160-250	2000 - 8000	0.4 - 0.8	*D/uL:* 0.8 - 1.6
50	0.5	1.5	11	0.78		500 - 6000	1.0	Axial dispersion (batch)
4.4		3.0	1.6	0.87		0 - 1600		Axial dispersion
5	9	3	1.6	0.87	10 - 58	100 - 1000	0.08 - 0.4	*D/uL* minimum when $Re_o = 100 - 300$

3. Full Description of Experimental Conditions and Data Fitting for Residence Time Distribution Studies

The full set of operating conditions for the RTD measurements performed in this work are listed in Table S2.

Table S2. Experimental conditions for residence time distribution studies.

Amplitude (mm)	Frequency (Hz)	Flow Rate (g/min)
38	1	50
38	1	50
38	1	200
38	1	200
66	1	50
66	1	50
66	1	200
66	1	200
9	3	50
9	3	50
30	3	200
30	3	200
14	3	50
14	3	50

The experimental data were fitted to a plug flow with axial dispersion model exploiting the imperfect pulse technique. A detailed explanation of this procedure is given here. The axial dispersion E, was obtained by solving the following equation:[15]

$$\sigma_\theta^2 = \frac{\sigma^2}{\tau^2} = 2\left(\frac{E}{uL}\right) - 2\left(\frac{E}{uL}\right)^2\left(1 - e^{-\frac{uL}{E}}\right)$$

where σ^2 is variance i.e., a measure of the spread of the RTD curve. The mean residence time, τ, was calculated from the experimental concentration and time data using:

$$\tau = \frac{\sum t_i C_i \Delta t_i}{\sum C_i \Delta t_i} = \frac{\sum t_i C_i}{\sum C_i}$$

where t_i is the experimental time with a given concentration, C_i. The variance, is calculated using:

$$\sigma^2 = \frac{\sum t_i^2 C_i \Delta t_i}{\sum C_i \Delta t_i} - \left[\frac{\sum t_i C_i \Delta t_i}{\sum C_i \Delta t_i}\right]^2 = \frac{\sum t_i^2 C_i}{\sum C_i} - \left[\frac{\sum t_i C_i}{\sum C_i}\right]^2$$

Given experimentally a perfect pulse of tracer cannot be achieved, the imperfect pulse method is applied to provide a more realistic description of the experimental tracer profile.

The imperfect pulse method for determining axial dispersion accounts for the finite dispersion of tracer concentration over the duration of the injection. The basic differential equation representing the dispersion model, used previously to describe dispersion in oscillatory flow mixing, is represented by:

$$\frac{\partial C}{\partial \theta} = \left(\frac{E}{uL}\right)\frac{\partial^2}{\partial Z^2} - \frac{\partial C}{\partial Z}$$

where C is the nomalised concentration; θ is dimensionless time, $\theta = ut/\mathrm{L}$; Z is dimensionless length, $\mathrm{Z} = x/\mathrm{L}$; u (m s^{-1}) is the mean axial velocity; L (m) is the length of reactor used; E (m^2 s^{-1}) is the axial dispersion coefficient; and t is time, measured from the tracer injection. This differential equation permits the prediction of pulse profile, from the calculated axial dispersion coefficient, downstream from the original measurement. This equation permits the prediction of a pulse profile, from the calculated axial dispersion coefficient, downstream from the original measurement.

The imperfect pulse model was fitted to experimental data, and completed using procedures described in detail elsewhere.[5] Concentrations were normalised using the following equation:

$$C(t) = \frac{c(t)}{\int_0^\infty c(t)dt} = \frac{C_{pulse}}{\frac{M}{v}} = \frac{C_i}{\sum C_i \Delta t_i}$$

The mass of tracer flowing through the system was calculated from the area under the RTD curve (summation of $C_i \Delta t_i$), where M is the mass of tracer (kg) and v is fluid velocity m^3 s^{-1}. This material balance allows confirmation of tracer recovery once the experiment is complete.

A transfer function, $TR(t)$, was applied to convert the upstream response to the downstream response, described by:

$$TR(t) = \sqrt{\frac{Pe\tau}{4\pi t^3}} exp\left\{\frac{-Pe}{\frac{4t}{\tau}}\left(1 - \frac{t}{\tau}\right)^2\right\}$$

Using the Pe number, the axial dispersion coefficient was calculated. The model response $C_2(t)'$ at position 2 was calculated by convolving the measured input response $C_1(t)'$ and the model transfer function $TR(t)$:

$$C_2(t)' = \int_0^t TR(t-p)C_1(p)\mathrm{d}p.$$

where p is the time response downstream and t is the time response from the upstream response. The model calculated response $C_2(t)'$ was then compared to the experimental $C_2(t)$ using the target function defined as:

$$\Delta C = \sum_{i=1}^{N} \{C_2(t_i) - C_2(t_i)'\}^2$$

The Pe number was varied to find the value resulting in minimal difference between the calculated and predicted responses, this value was taken as the Pe number of the system. A representative result obtained using this fitting procedure is shown in Figure S2. Using the Pe number obtained from the minimal difference between predicted and observed values, an overlay of the raw data with the predicted response can be plotted (Figure S2, right panel).

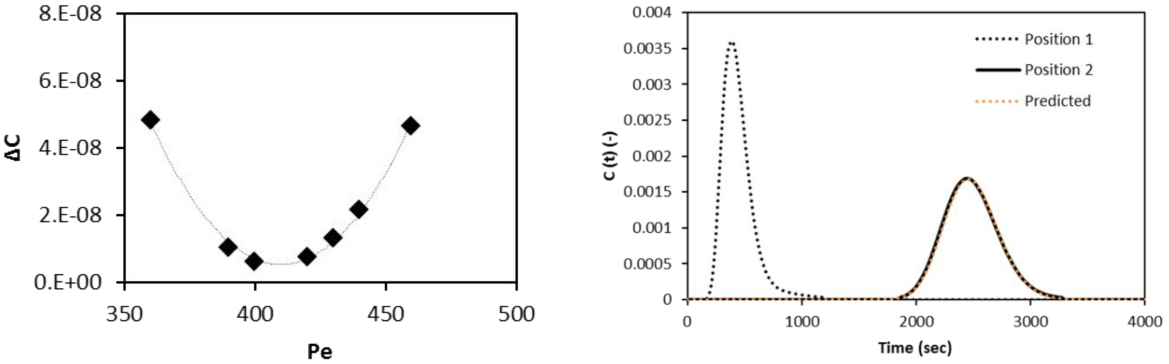

Figure S2. Application of Peclet number. Difference plot (left) used to determine the Peclet number, and representative concentration data (right, dotted and solid black; 100 g/min, 30 mm amplitude, 1 Hz), with the model response overlaid (orange).

4. Full Experimental Conditions and Temperature Data for Heat Transfer Studies

The full set of experimental conditions for the heat transfer studies are given in Table S3.

Table S3. Experimental conditions for heat transfer studies.

Oscillation Setup	Solution Mass Flow Rate (g/min)	Jacket Mass Flow Rate (g/min)
Single	50	50
Single	50	250
Single	50	500
Single	50	750
Single	50	1000
Single	50	1700
Single	50	2700
Single	100	100
Single	150	150
Single	200	200
Single	200	1700
Single	200	2700
Double	50	25
Double	50	50
Double	100	100
Double	150	75
Double	150	81
Double	150	89
Double	150	150
Double	150	162
Double	200	200
Double	200	220
Double	200	240

Double	200	280

The full experimental temperature data obtained from single and double oscillations setups can be found in Figure S3 and Figure S4 respectively.

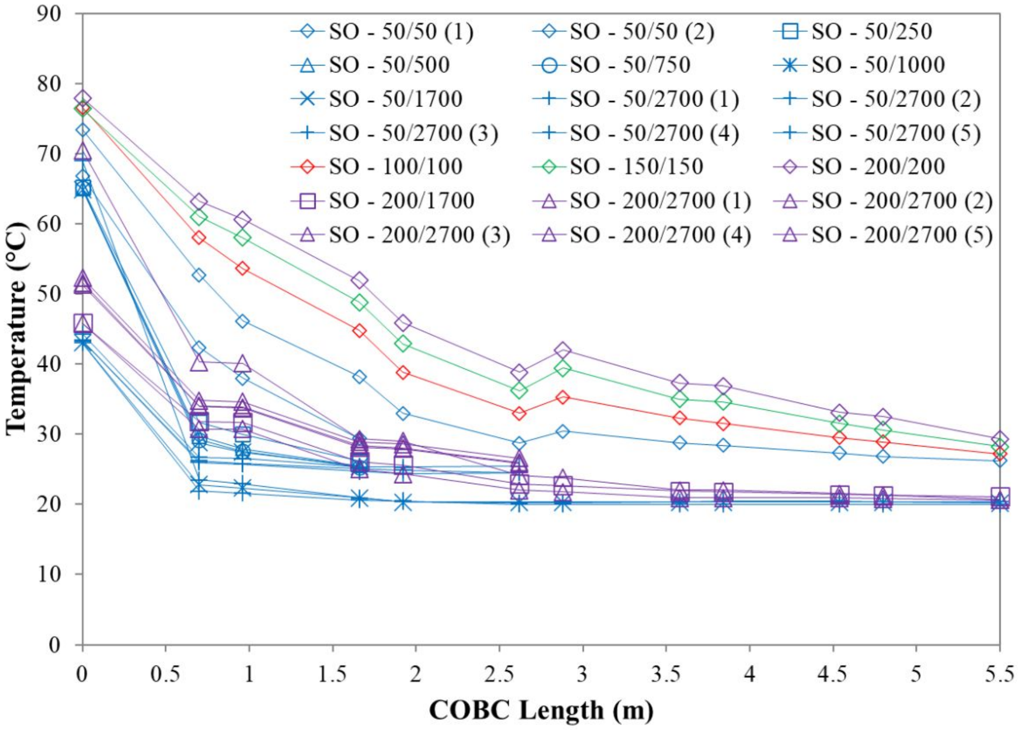

Figure S3. Experimental temperature data for all single oscillation experiments. The data have been connected by lines as a guide (lines do not represent the actual profile).

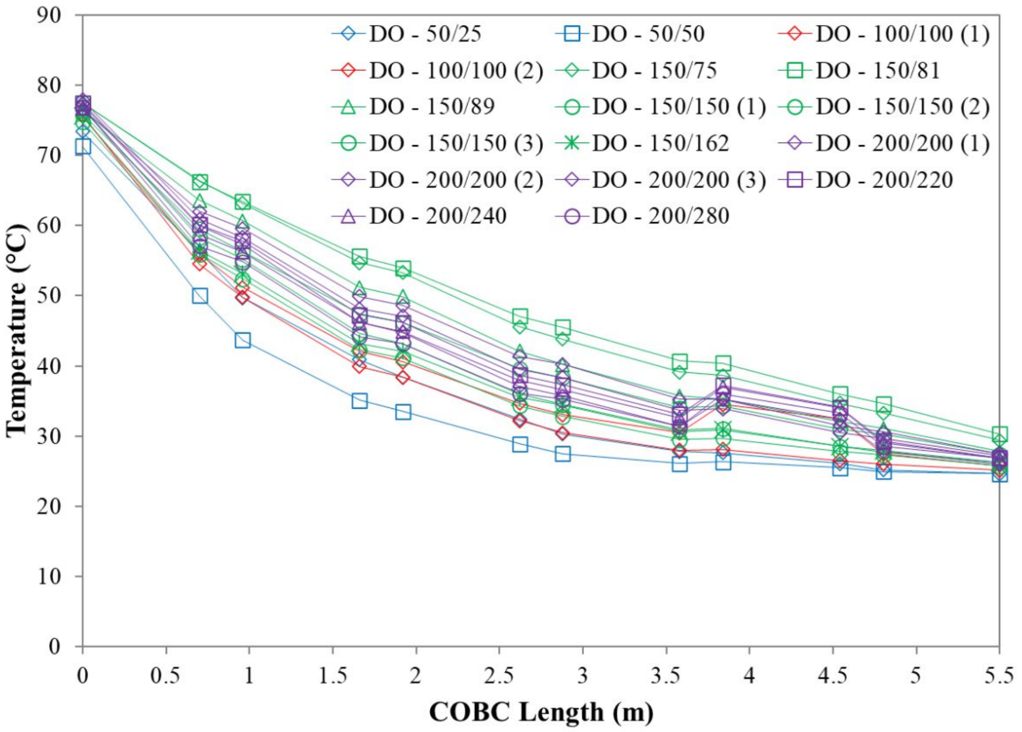

Figure S4. Experimental temperature data for all double oscillation experiments. The data have been connected by lines as a guide (lines do not represent the actual profile).

5. Full Description of Temperature Profile Model

A method for determining the overall heat transfer coefficient (U) for oscillatory flow in baffled tubes from experimental temperature data has been outlined previously.[16] Firstly, it considers that U can be determined from the following equation for the heat transfer rate (Q):

$$Q = A\ U\ \Delta T_{lm} = Q_{sol} = m_1\ c_p\ \Delta T_{sol}$$

Secondly, it considers that the log mean temperature difference (ΔT_{lm}) over a COBC section can be given by:

$$\Delta T_{lm} = \frac{\Delta T_2 - \Delta T_1}{ln\left(\frac{\Delta T_2}{\Delta T_1}\right)}$$

$$\Delta T_1 = T_{In} - T_2$$

$$\Delta T_2 = T_{Out} - T_1$$

By substituting the expression for the log mean temperature difference into the expression for Q, the overall heat transfer coefficient for a COBC section can be determined by the following equation:

$$U = \frac{m_1\, c_{p1}\, \Delta T_{sol}}{A\, \Delta T_{lm}}$$

$$A = \pi\, d_t\, L$$

The values in these expressions are the mass flow rate of the solution (m_1); the specific heat capacity of the solution (c_{p1}); the total heat transfer area of the COBC section based on its inner diameter (A); the inlet and outlet temperatures of the solution (T_{In} and T_{Out}); the inlet and outlet temperatures of the jacket fluid (T_1 and T_2), the temperature difference in the solution (ΔT_{sol}) and the temperature difference in the jacket fluid (ΔT_j).

According to Newton's law of cooling/heating, the temperature of the solution (T_1) will vary along the COBC length (x) according to the following differential equation:

$$\frac{dT_1}{dx} = k_1(T_2 - T_1)$$

When the solution flows through a straight T_2 represents the temperature of the jacket fluid and when the solution flows through an unjacketed bend T_2 represents the temperature of the surrounding air. Assuming plug flow in the COBC, the constant (k_1) in the differential equation is given by:

$$k_1 = \frac{U_1\, a_1\, A_{xs1}}{m_1\, c_{p1}}$$

$$a_1 = {}^{4}/{d_t}$$

$$A_{xs1} = \pi\, {d_t}^2/4$$

The values in these expressions are the heat exchange area per unit volume (a_1), the cross-sectional area based on the inner diameter (A_{XS1}), the mass flow rate of the solution (m_1) and the specific heat capacities of the solution (c_{p1}).

Using the overall heat transfer coefficients, and making certain assumptions, the temperature profile along the COBC can be modelled. Depending on which assumptions are made, the temperature profile model can have three levels. Level 1 assumes that the jacket fluid

temperature is constant and that there are no heat losses from the jacket. Level 2 assumes that the jacket fluid varies and that there are no heat losses from the jacket. Level 3 assumes that the jacket fluid varies and that there are heat losses from the jacket. For each level, the relevant differential equations must be solved either numerically or analytically in order to model the temperature profile along the COBC.

Level 1: Constant Jacket Fluid Temperature, No Losses

Differential Equations

$$\frac{dT_1}{dx} = k_1(T_2 - T_1)$$

$$\frac{dT_2}{dx} = 0$$

$$k_1 = \frac{U_1\, a_1\, A_{xs1}}{m_1\, c_{p1}}$$

General Analytical Solution

$$T_1(x) = T_2 + (T_{10} - T_2)\, e^{-k_1 x}$$

$$T_2(x) = T_2$$

Level 2: Variable Jacket Fluid Temperature, No Losses

Differential Equations

$$\frac{dT_1}{dx} = k_1(T_2 - T_1)$$

$$\frac{dT_2}{dx} = -\, k_2(T_1 - T_2)$$

$$k_1 = \frac{U_1\, a_1\, A_{xs1}}{m_1\, c_{p1}}$$

$$k_2 = \frac{U_1\, a_1\, A_{xs1}}{m_2\, c_{p2}}$$

General Analytical Solution

$$T_1(x) = C_1 + C_2\, e^{(-k_1 + k_2)x}$$

$$T_2(x) = \frac{C_2\, e^{(-k_1 + k_2)x}\, k_2}{k_1} + C_1$$

$$C_1 = \frac{T_{20} - T_{10}\left(\frac{k_2}{k_1}\right)}{1 - \left(\frac{k_2}{k_1}\right)}$$

$$C_2 = T_{10} - C_1$$

Level 3: Variable Jacket Fluid Temperature, Losses

Differential Equations

$$\frac{dT_1}{dx} = k_1(T_2 - T_1)$$

$$\frac{dT_2}{dx} = -\,k_2T_1 + (k_2 + k_3)T_2 - k_3T_a$$

$$k_1 = \frac{U_1\,a_1\,A_{xs1}}{m_1\,c_{p1}}$$

$$k_2 = \frac{U_1\,a_1\,A_{xs1}}{m_2\,c_{p2}}$$

$$k_3 = \frac{U_2\,a_2\,A_{xs2}}{m_2\,c_{p2}}$$

$$a_2 = {}^4/(d_s - d_t)$$

$$A_{xs2} = \left(\pi\,d_s{}^2/4\right) - \left(\pi\,d_t{}^2/4\right)$$

General Analytical Solution

$$T_1(x) = e^{(S\,+\,R)x}\,C_2 + e^{(S\,-\,R)x}\,C_1 + T_a$$

$$T_2(x) = \frac{1}{k_1}\left[(S + R)\,e^{(S\,+\,R)x}\,C_2 + (S - R)\,e^{(S\,-\,R)x}\,C_1 + k_1\left(e^{(S\,+\,R)x}\,C_2 + \,e^{(S\,-\,R)x}\,C_1 + T_a\right)\right]$$

$$C_1 = \frac{T_{20}k_1 - T_{10}[S + R + k_1] - T_a[\,-S - R]}{-2R}$$

$$C_2 = T_{10} - T_a - C_1$$

$$S = \frac{1}{2}(\,-k_1 + k_2 + k_3)$$

$$R = \frac{1}{2}\sqrt{k_1{}^2 - 2\,k_1\,k_2 + 2\,k_1\,k_3 + k_2{}^2 + 2\,k_2\,k_3 + k_3{}^2}$$

The extra terms which arise in levels 2 & 3 of the temperature profile model are, m_2 = mass flow rate of jacket fluid (kg s^{-1}), c_{p2} = specific heat capacity of jacket fluid (J/kg °C), d_s = inner diameter of the shell (m), a_2 = the heat exchange area per unit volume with respect to the shell (m^{-1}), A_{xs2} = cross-sectional area with respect to the shell (m^2), T_{10} = initial temperature of solution (°C), T_{20} = initial temperature of jacket fluid (°C), T_a = air temperature (°C), k_1,k_2 & k_3 = differential equation constants (-), C_1 & C_2 = integration constants (-), and R & S = substitution constants (-).

6. Temperature and Supersaturation Profile Modelling for Crystallisation Process Design in the Continuous Oscillatory Baffled Crystalliser

This case study looks at the seeded cooling crystallisation of paracetamol in a Water/IPA (60/40 wt%) solvent mixture as a continuous process in the DN15 COBC. The assumptions for this case study are as follows:

- It is a seeded crystallisation process (no nucleation, only growth)
- The seed crystals are cubic, the same size and grow equally in all directions
- Growth rate constant and growth order are independent of crystal size and temperature
- The process is operating under plug flow conditions
- A counter-current cooling system is being utilised
- The jacket temperature remains constant

In order to implement the model, the solubility of paracetamol in this solvent system must be known over the temperature range which is explored. Solubility data for this system have been obtained previously[17] and are plotted in Figure S5.

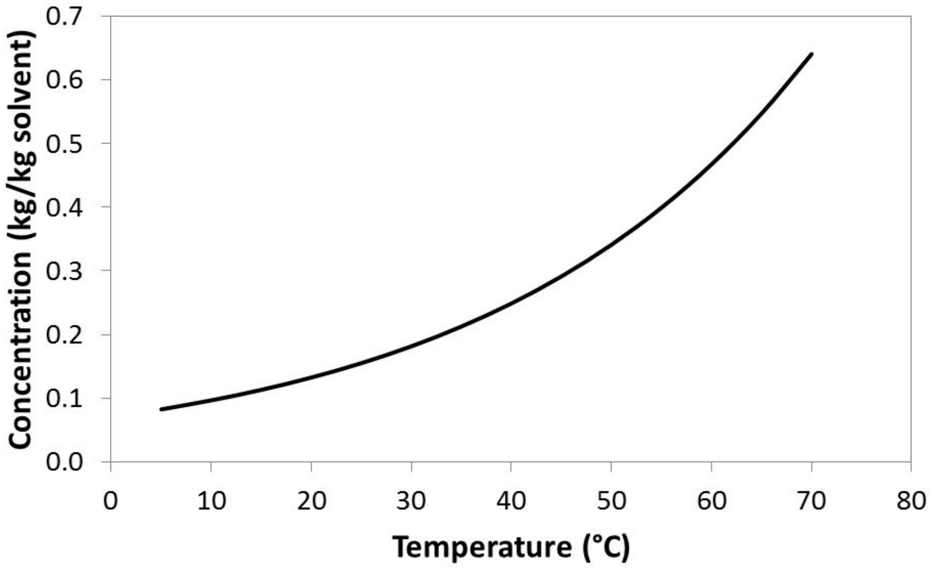

Figure S5. Experimental solubility of paracetamol in water/IPA (60/40 wt%).

The equation used to fit this solubility curve is as follows:

$$C_S = 0.0706\, e^{0.0315\, T_1}$$

The experimental procedure involves cooling a saturated paracetamol solution from 70 °C to a certain extent over a fixed COBC length (5.5m). Utilising a particular overall heat transfer coefficient (cooling method) will result in the solution being cooled to a particular final temperature over the COBC length. Using a basis of 1 kg solvent, the values of the constants involved in modelling the temperature and supersaturation profiles throughout the crystallisation process are listed in Table S4.

Table S4. Full list of constants involved in modelling the temperature and supersaturation profiles.

**Constant**	**Symbol (Unit)**	**Value**
Growth rate constant	k_g (m/s)	1 x 10^{-6}
Growth order	n (-)	1.5
Solvent mass	m_s (kg)	1
Total mass of API	m_t (kg)	0.6404
Initial seed loading	m_{x0} (wt%)	1
Crystal density	ρ_c (kg/m^3)	1260

Initial diameter of seeds	d (μm)	20
Solution mass flow rate	m_1 (g/min)	50
Specific heat capacity	c_p (J/kg °C)	4180
Area per unit volume	a_1 (m^{-1})	266.7
Cross-sectional area	A_{xs1} (m^2)	0.000177
Initial solution temperature	T_{10} (°C)	70
Jacket temperature	T_2 (°C)	20
Solution velocity	v (m/s)	0.004716
Overall heat transfer coefficient (1)	U_1 (W/m^2 K)	25
Overall heat transfer coefficient (2)	U_2 (W/m^2 K)	100
Overall heat transfer coefficient (3)	U_3 (W/m^2 K)	400

The variables which will change over the course of the crystallisation process as they are being determined by the model are given in Table S5.

Table S5. List of variables determined by the model.

**Variable**	**Symbol (Unit)**
Mass of API in solid phase	m_x (kg)
Solution temperature	T_1 (°C)
Solution concentration	C (kg/kg solvent)
Saturation concentration	C_s (kg/kg solvent)
Relative supersaturation	S (-)

References

1. Sobey, I. J., Flow through furrowed channels. Part 1. Calculated flow patterns. *J. Fluid Mech.* **1980,** 96, 1-26.
2. Sobey, I. J., Oscillatory flows at intermediate Strouhal number in asymmetric channels. *J. Fluid Mech.* **1982,** 125, 359-373.
3. Ni, X. W.; Pereira, N. E., Parameters affecting fluid dispersion in a continuous oscillatory baffled tube. *AIChE J.* **2000,** 46, (1), 37-45.
4. Stonestreet, P.; Van der Veeken, P. M. J., The effects of oscillatory flow and bulk flow components on residence time distribution in baffled tube reactors. *Chem. Eng. Res. Des.* **1999,** 77, (A8), 671-684.
5. Zheng, M. Z.; Mackley, M., The axial dispersion performance of an oscillatory flow meso-reactor with relevance to continuous flow operation. *Chem. Eng. Sci.* **2008,** 63, (7), 1788-1799.
6. Reis, N.; Vincente, A. A.; Teixeira, J. A.; Mackley, M. R., Residence times and mixing of a novel continuous oscillatory flow screening reactor. *Chem. Eng. Sci.* **2004,** 59, (22-23), 4967-4974.
7. Ni, X. W., A study of fluid dispersion in oscillatory flow-through a baffled tube. *J. Chem. Technol. Biotechnol.* **1995,** 64, (2), 165-174.
8. Mackley, M. R.; Ni, X., Mixing and dispersion in a baffled tube for steady laminar and pulsatile flow. *Chem. Eng. Sci.* **1991,** 46, (12), 3139-3151.
9. Dickens, A. W.; Mackley, M. R.; Williams, H. R., Experimental residence time distribution measurements for unsteady-flow in baffled tubes. *Chem. Eng. Sci.* **1989,** 44, (7), 1471-1479.
10. Howes, T.; Mackley, M. R., Experimental axial-dispersion for oscillatory flow through a baffled tube. *Chem. Eng. Sci.* **1990,** 45, (5), 1349-1358.
11. Mackley, M. R.; Ni, X., Experimental fluid dispersion measurements in periodic baffled tube arrays. *Chem. Eng. Sci.* **1993,** 48, (18), 3293-3305.
12. Ni, X. W., Residence time distribution measurements in a pulsed baffled tube bundle. *J. Chem. Technol. Biotechnol.* **1994,** 59, (3), 213-221.
13. Mackley, M. R.; Stonestreet, P.; Roberts, E. P. L.; Ni, X., Residence time distribution enhancement in reactors using oscillatory flow. *Chem. Eng. Res. Des.* **1996,** 74, (A5), 541-545.

14. Fitch, A. W.; Ni, X., On the determination of axial dispersion coefficient in a batch oscillatory baffled column using laser induced fluorescence. *Chem. Eng. J.* **2003,** 92, (1-3), 243-253.

15. Levenspiel, O., Chemical reaction engineering. *Ind. Eng. Chem. Res.* **1999,** 38, (11), 4140-4143.

16. Mackley, M. R.; Stonestreet, P., Heat-transfer and associated energy-dissipation for oscillatory flow in baffled tubes. *Chem. Eng. Sci.* **1995,** 50, (14), 2211-2224.

17. Hojjati, H.; Rohani, S., Measurement and prediction of solubility of paracetamol in water-isopropanol solution. Part 1. Measurement and data analysis. *Org. Process Res. Dev.* **2006,** 10, (6), 1101-1109.
